# Disease-specific gut microbiota–metabolite–immune networks in hematologic disorders: mechanisms, causal support, and translational strategies

**DOI:** 10.1080/19490976.2026.2752469

**Published:** 2026-10-29

**Authors:** Li Yi, Yuru Yuan, Xiaolong Ma, Chenxi Li, Yushuo Hao, Qianran Ma, Xuexue Liu, Xiao Du, Lihua Shao, Siliang Wang, Jingyan Xu

**Affiliations:** a Department of Hematology, Nanjing Drum Tower Hospital Clinical College of Nanjing University of Chinese Medicine, Nanjing, China; b School of Life Sciences, Nanjing Normal University, Nanjing, China; c Department of Colorectal Surgery, Nanjing Drum Tower Hospital, Affiliated Hospital of Medical School, Nanjing University, Nanjing, China; d Department of Pharmacy, Nanjing Drum Tower Hospital Clinical College of Nanjing University of Chinese Medicine, Nanjing, China; e Department of Thyroid, Metabolic and Hernia Surgery, The First Affiliated Hospital of Dalian Medical University, Dalian, China; f Department of Pharmacy, Nanjing Drum Tower Hospital, Affiliated Hospital of Medical School, Nanjing University, Nanjing, China; g Department of Hematology, Nanjing Drum Tower Hospital, Affiliated Hospital of Medical School, Nanjing University, Nanjing, China

**Keywords:** Gut microbiota, hematologic disorders, microbial metabolites, host–microbiome interactions, bone marrow niche, immune remodeling, causal support, translational microbiome medicine

## Abstract

The gut microbiota is increasingly recognized as a systemic regulator of host immunity, host metabolism, epithelial barrier integrity, and distal tissue homeostasis. Hematologic disorders provide an important setting in which these host–microbe interactions converge on immune remodeling, marrow-niche function, hematopoietic recovery, treatment response, and transplant-related complications. This narrative review synthesizes mechanistic, clinical, and translational evidence linking gut microbial dysbiosis to benign and malignant hematologic diseases, clonal and premalignant states, and hematopoietic stem cell transplantation (HSCT)-related outcomes. Rather than treating microbiome associations as a single evidence category, we distinguish five complementary evidence strata: Mendelian randomization, experimental models, prospective human cohorts, randomized human interventions, and cross-sectional studies. These study designs address different causal questions and are interpreted as complementary rather than as a single linear hierarchy. We also separate microbial composition from microbial function: taxon-level enrichment or depletion is treated primarily as context-dependent and hypothesis-generating, whereas conserved functional pathways—including short-chain fatty acids, bile-acid transformation, tryptophan metabolism, microbial translocation and barrier dysfunction, and innate immune activation—provide the principal cross-disease mechanistic framework. We integrate disease-specific microbial features, functional mediators, immune and marrow consequences, and clinical outcomes into an author-constructed evidence map, while explicitly addressing bidirectionality, reverse causality, and translational readiness. Emerging applications include microbiota-based biomarkers, treatment-response prediction, fecal microbiota transplantation, probiotics and postbiotics, antibiotic stewardship, and microbiota-dependent modulation of drug metabolism.

## 1. Introduction and evidence framework

Hematologic diseases are increasingly understood as disorders shaped by both cell-intrinsic lesions and systemic host-environment interactions. High-throughput sequencing and systems-biology approaches have clarified pathogenic networks in leukemia, lymphomas, myelodysplastic syndromes, and marrow-failure states.^1-4^ Yet several layers of regulation remain insufficiently integrated, particularly pathway-level host–microbe interactions, genotype–phenotype coupling, and environmental inputs that may modify hematologic risk, immune tone, or therapeutic response.

The hematologic system is especially responsive to systemic microbial and metabolic cues because it maintains continuous blood-cell production, host defense, and circulation of bioactive mediators.^5^ Leukemias, lymphomas, myelodysplastic syndromes, marrow-failure states, and post-transplant immune complications may therefore reflect not only cell-intrinsic mutations or local niche injury but also long-range tissue-to-tissue communication. Classical models emphasized the marrow microenvironment; more recent work has expanded this view toward immune–oncology–microbiota interactions and multi-organ host–microbe crosstalk.^6^ ^,^ ^7^

Within this framework, the gut microbiota functions as a systemic source of immune, metabolic, and barrier-derived signals. Short-chain fatty acids (SCFAs), bile acids, lipopolysaccharide, peptidoglycan, tryptophan metabolites, and other microbial products regulate host immunity through pattern-recognition receptors, metabolic receptors, epigenetic mechanisms, and epithelial-barrier pathways. These signals can shape Treg/Th17 balance, hematopoietic stem and progenitor cell (HSPC) function, myeloid inflammatory skewing, and immune-cell differentiation across compartments far beyond the intestine.^8-11^

The field is methodologically uneven. Mendelian randomization studies, germ-free or genetically modified animal models, prospective cohorts, cellular experiments, and small cross-sectional association studies answer different questions and provide different levels of causal inference. We therefore treat causal support as a property of the study design and biological chain being tested, rather than as a single label applied uniformly across diseases. On that basis, this review distinguishes relatively strong causal evidence, mechanistic or prospective support, and preliminary association-based findings, while also considering how each evidence type may or may not translate into microbiota-oriented intervention.

We structure the review around four microbiome-centered questions: (i) how gut microbial signals reach the hematopoietic and marrow-niche system; (ii) how dysbiosis differs across hematologic phenotypes, from benign cytopenias to malignant disease; (iii) how microbial metabolites and products reprogram T-cell, B-cell, and myeloid compartments; and (iv) how these findings may be translated into biomarkers, microbiota-targeted interventions, antibiotic stewardship, and treatment-response prediction.

Because the gut microbiota–hematology literature is methodologically heterogeneous, we assess the evidence along two complementary axes: study design and causal inference, and microbial composition and function.

The first axis comprises five evidence strata. Mendelian randomization (MR) assesses the potential direction of a causal effect at the population level but does not establish mechanism; experimental models, including germ-free or gnotobiotic systems, genetically modified models, defined-colonization and microbiota-transfer studies, and related mechanistic experiments, can establish biological necessity or molecular pathways in controlled systems but cannot by themselves establish causality in humans; prospective human cohorts provide temporal information by relating baseline microbial features to subsequent outcomes but remain susceptible to confounding; randomized human interventions directly test whether deliberate modification of the microbiota or related pathways changes a predefined outcome and provide the most direct evidence of clinical modifiability, although such studies remain limited in hematology; cross-sectional studies characterize disease-associated microbial patterns but cannot establish temporal direction.

These strata are complementary rather than a single linear hierarchy because they address different causal questions. Accordingly, findings are interpreted according to the inference permitted by the study design and are not combined into a single causal claim. Non-randomized interventions and case reports are not considered equivalent to randomized trials and are interpreted according to their design and endpoints. Methodologically, similar triangulation has been applied in oncology by combining replicated bidirectional MR with immune-trait mediation analysis to interrogate candidate gut microbiota–immune cell–glioma pathways, illustrating how causal direction and potential immune mediators can be evaluated without treating genetic association as proof of mechanism.^12^

The second axis separates microbial composition from function. Taxonomic findings are strongly influenced by cohort, geography, diet, medication, and treatment exposure and are therefore treated primarily as hypothesis-generating unless supported by functional evidence. Functional evidence is considered at the level of conserved microbial pathways or products with measurable host effects, including short-chain fatty acids (SCFAs), bile-acid transformation, tryptophan metabolism, microbial translocation and epithelial barrier dysfunction, and innate immune activation through pattern-recognition receptors. These pathways provide the cross-disease mechanistic framework. Where compositional and functional findings coexist, their concordance and the level of causal support are assessed explicitly.

This framework distinguishes causal direction, mechanism, temporal association, and clinical modifiability. A finding is therefore reported only at the level supported by its design. Where evidence supports more than one direction, particularly for CHIP, MPN, leukemia, and HSCT, the relationship is described as bidirectional or directionally unresolved Table 1.

**Table 1.** Cross-disease evidence map for gut microbiota–hematology research: evidence strata, causal limitations, and translational potential.

Disease/clinical setting	Evidence strata	Representative evidence	Main limitation	Translational potential	Priority next step
Basic hematopoietic regulation	EXP	Gnotobiotic, microbiota-depletion, and recolonization models^13-16^	Marked differences between murine and human microbiota; no human dose–response data for SCFAs; causal direction between hematopoietic abnormalities and dysbiosis remains uncertain	M	Longitudinal cohorts; humanized microbiota models; human pharmacokinetic and exposure–response studies of microbial metabolites
Anemia	EXP/RCT	Microbial iron-homeostasis models; randomized renal-anemia dietary-fiber intervention^17^ ^,^ ^18^	Substantial subtype heterogeneity; bidirectional iron–microbiota causality is difficult to disentangle; clinical intervention data are largely confined to renal anemia and surrogate/hematologic endpoints	M	Multicenter prospective intervention studies stratified by anemia subtype
ITP	MR/PRO/CS	MR; prospective microbiota-based corticosteroid-response study; cross-sectional microbiome/metabolome evidence^19-21^	Prediction models lack multicenter external validation; temporality of gut microbiota change is unclear; specificity of metabolite mediators remains to be confirmed	M	Longitudinal designs in newly diagnosed, treatment-naïve patients
MDS	MR/CS	MR; cross-sectional and treatment-associated human studies^22-24^	Small samples; major subtype heterogeneity; lack of pre-diagnostic baseline data	M	Multicenter longitudinal cohorts with pre-diagnostic or early-disease sampling
MPN/CHIP	MR/EXP/RCT/CS	MR; experimental models; randomized dietary intervention; cross-sectional microbiome studies^25-29^	Different study designs address different causal directions; MPN MR instruments/case numbers are limited; longitudinal human gut-microbiome data remain scarce	M	Longitudinal cohorts linking microbiome, clonal burden, infection, and inflammatory phenotypes; mechanistic validation and microbiota-directed intervention
Leukemia	EXP/PRO/CS	Experimental leukemia models; prospective and cross-sectional human studies^30-33^	Animal models are genetically restricted (e.g., ETV6-RUNX1); antibiotic and chemotherapy effects are difficult to separate; diagnostic models lack cross-disease validation	M	Multicenter validation cohorts; clinical designs able to disentangle antibiotics from chemotherapy
Lymphoma	MR/EXP/PRO/CS	MR; experimental models; prospective and cross-sectional human studies^34-39^	Directionality and taxonomic signatures differ across subtypes; external validation is limited; some follicular-lymphoma evidence remains conference-abstract level	M	Large multicenter cohorts rigorously stratified by subtype
Multiple myeloma	EXP/CS	Clinical association/validation cohorts; ex vivo, in vitro, and in vivo mechanistic studies^40^ ^,^ ^41^	Human pharmacokinetics of urolithin A are lacking; treatment-response associations remain vulnerable to confounding; longitudinal precursor-state data are limited	L	Longitudinal cohorts spanning MGUS/SMM-to-MM progression; phase I pharmacokinetic and safety studies of urolithin A
CLL	EXP/CS	Eμ-TCL1 mouse model; cross-sectional cohorts^42^ ^,^ ^43^	More than 90% of gut microbiota variance remains unexplained; known determinants such as disease status and medication explain only a small fraction; temporality and directionality remain unclear	L	Prospective longitudinal sampling in slowly progressive CLL
HSCT-related complications	EXP/PRO/RCT	Mechanistic studies; prospective multi-omics cohorts; randomized FMT trial^44-48^	Microbiome signals are strongly confounded by conditioning, antibiotics, nutrition, and disease severity; randomized FMT improved ecological recovery without significantly reducing infections; optimal timing and long-term safety remain uncertain	H	Multicenter randomized trials powered for GVHD, infection, relapse, and survival; define timing and formulation of microbiota restoration
Immune regulation (CAR-T/ICI)	EXP/PRO	Prospective clinical cohorts; animal validation^49-51^	Antibiotic exposure is confounded by infection severity and treatment indication; microbiota-response models need multicenter prospective validation	M	Prospective studies testing whether microbiota-informed or microbiota-directed strategies improve treatment efficacy or toxicity
Clinical translation	RCT	Randomized FMT trial with additional non-randomized or retrospective microbiota-restoration studies^48^ ^,^ ^52^ ^,^ ^53^	Microbiome restoration does not necessarily translate into clinical benefit; long-term FMT safety and microbiome-testing standardization remain unresolved	M	Standardized microbiome testing and multicenter interventional trials with clinically meaningful hematologic endpoints

Note: This author-constructed map is intended to compare disease areas within the gut microbiota–hematology interface and should not be interpreted as a formal GRADE assessment. Evidence strata are denoted by study-design abbreviations rather than ranked grades: MR, Mendelian randomization; EXP, experimental/mechanistic models (including germ-free/gnotobiotic, genetic, defined-colonization, microbiota-transfer, and related mechanistic studies); PRO, prospective human cohorts; RCT, randomized human interventions; CS, cross-sectional studies. Non-randomized interventions, retrospective treatment cohorts, and case reports are described separately and are not treated as equivalent to RCT evidence. Translational potential: H (high) = sufficient evidence to justify early interventional clinical-study design; M (moderate) = additional mechanistic validation, longitudinal confirmation, or sample expansion is still required; L (low) = currently hypothesis-generating or exploratory. FMT, fecal microbiota transplantation; GVHD, graft-versus-host disease; HSCT, hematopoietic stem cell transplantation; MM, multiple myeloma; ITP, immune thrombocytopenia; MDS, myelodysplastic syndromes; MPN, myeloproliferative neoplasms; CHIP, clonal hematopoiesis of indeterminate potential; CLL, chronic lymphocytic leukemia.

To make comparisons across diseases more transparent, we apply a two-dimensional framework that considers both causal support and translational potential. The framework is not meant to impose a conventional evidence-based-medicine hierarchy; rather, it asks how far a given body of work has progressed from association to mechanism and from mechanism to clinical tractability. After this cross-disease overview, Figure 1 synthesizes disease-stratified microbiota features, microbial metabolites or signals, immune and marrow-niche consequences, and clinical or biological outcomes. The figure highlights a central point of the review: gut microbiota–related mechanisms recur across hematologic disorders, but they do not form a single uniform pathway.

**Figure 1.**
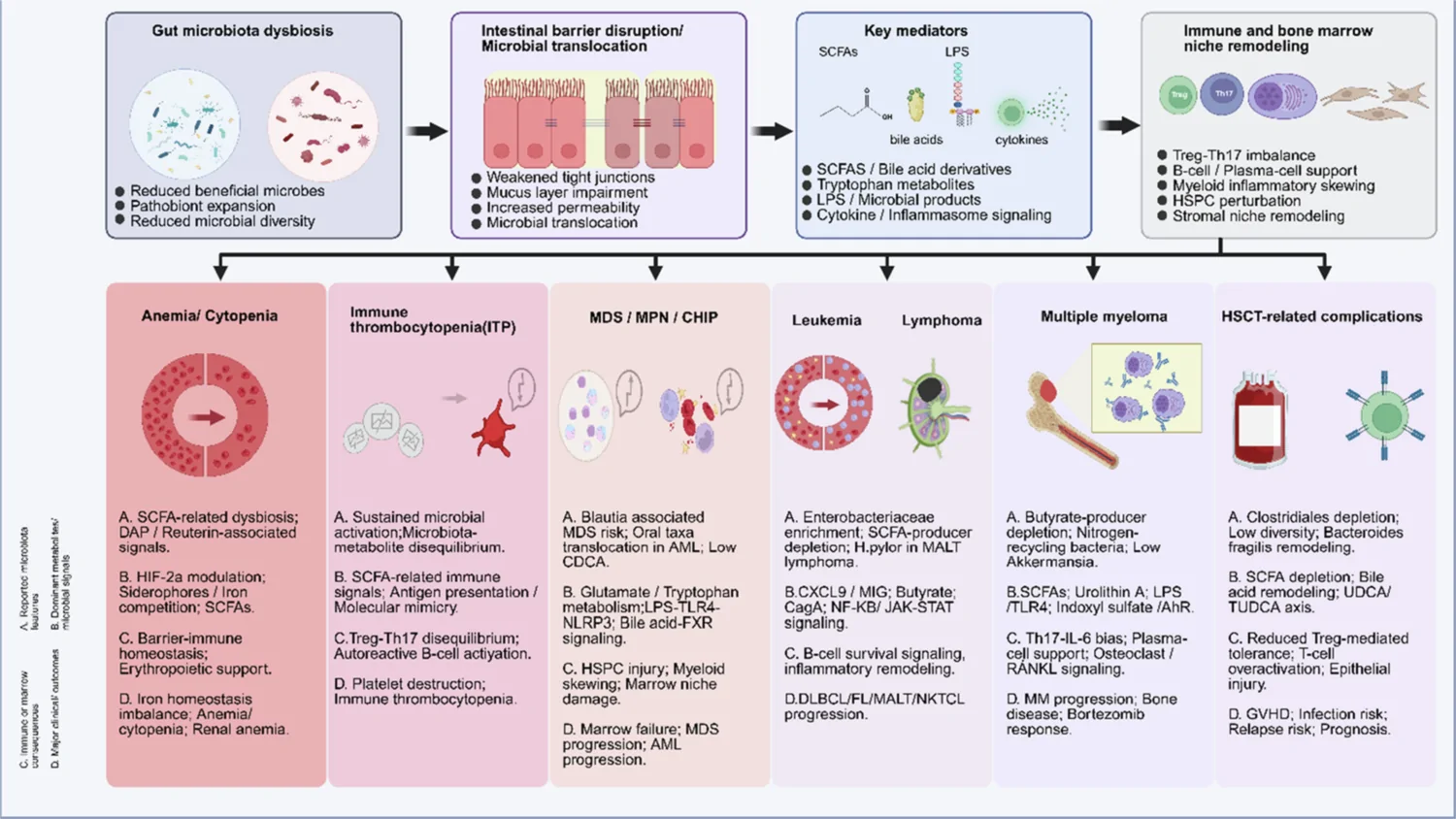
Representative disease-specific mechanisms linking gut microbiota dysbiosis to hematologic disorders. The schematic combines a shared upstream mechanistic axis with selected disease-specific branches. The upper layer summarizes recurring events, including microbial dysbiosis, barrier disruption, microbial translocation, altered microbial metabolites or products, immune remodeling, HSPC perturbation, and bone marrow niche changes. The lower layer illustrates representative patterns across anemia/cytopenia, immune thrombocytopenia, MDS/AML, leukemia, lymphoma, multiple myeloma, and HSCT-related complications, showing reported microbiota features, dominant metabolites or microbial signals, immune or marrow consequences, and major clinical or biological outcomes. The figure underscores that gut microbiota-related hematologic mechanisms are disease-specific networks rather than interchangeable variants of a single pathway.

## 2. Clinical associations between the microbiota and hematologic diseases

### 2.1. Microbiome profiles and the biological basis of hematopoietic regulation

In healthy individuals, bacteria, fungi, and viruses inhabit the gut, skin, lung, and oral cavity and contribute to nutrient metabolism, immune education, vitamin synthesis, and colonization resistance.^54^ ^,^ ^55^ The wider host-associated ecosystem also includes viral communities whose composition varies with host genetic and environmental factors.^56^ Although the present review focuses on the gut microbiota, this compartment should be considered part of a wider host–microbe ecosystem. Under physiological conditions, community composition changes with age, diet, medications, and environmental exposure, yet functional balance is usually preserved.

#### 2.1.1. Hematopoietic regulation

Gut-derived cues regulate hematopoiesis across the life course. During embryonic development, commensal microbiota support HSPC formation and differentiation by tuning basal inflammatory cytokine expression within the hematopoietic niche.^13^ In adults, microbiota-dependent innate signaling and microbial metabolites can influence HSC maintenance and lymphoid–myeloid output, although individual pathways remain strongly context dependent.^57^ ^,^ ^58^ These observations support a role for gut-derived signals in hematopoietic homeostasis without implying that a single microbial pathway governs all blood-cell lineages.

Microbial signals are also relevant during recovery from hematopoietic injury. In murine models, depletion of SCFA-producing bacteria impairs recovery from chemotherapy-induced myelosuppression and increases damage in long-term HSPCs.^14^ In transplantation, sustained microbiota injury and broad-spectrum antibiotic exposure are associated with adverse immune-reconstitution and transplant outcomes, although their causal contribution is difficult to isolate from conditioning, infection, and other clinical exposures.^59^

The most compelling mechanistic data for microbiota-dependent hematopoietic regulation still come from gnotobiotic, microbiota-depletion, recolonization, and related experimental models.^13-16^ Direct extrapolation to patients is not straightforward: murine and human microbiotas differ substantially, human studies rarely establish whether dysbiosis is cause or consequence, and dose–response data for microbial metabolites such as SCFAs are sparse. Longitudinal human cohorts paired with humanized models are therefore needed before clinically meaningful exposure thresholds can be defined.

#### 2.1.2. Trained immunity and basal defense

Trained immunity provides a conceptual framework for durable innate immune reprogramming by environmental and microbial cues. It denotes persistent changes in innate responsiveness driven by epigenetic and metabolic remodeling in cells such as monocytes, macrophages, and NK cells.^60^ Gut-derived SCFAs can alter histone acetylation and immune-cell differentiation, while experimental depletion of SCFA-producing bacteria can leave persistent chromatin and transcriptional changes in hematopoietic precursors.^14^ ^,^ ^61^ Together, these observations provide a plausible epigenetic link between microbial exposure and durable hematopoietic or immune states, but they do not establish that SCFAs uniformly induce a protective trained-immunity program in patients.

SCFAs signal through G-protein–coupled receptors and HDAC inhibition to regulate immune-cell differentiation and inflammatory tone.^61^ ^,^ ^62^ Indole derivatives can activate aryl hydrocarbon receptor (AhR) signaling, induce IL-22, and support epithelial regeneration and barrier integrity, an axis of potential relevance during intensive AML chemotherapy.^63^ In adaptive immunity, microbial metabolites can influence Treg, Th17, and CD8+ T-cell programs, but the direction and magnitude of these effects depend on cell type, metabolite concentration, and disease context.^61^

Two features of this literature limit how far it can be applied clinically. First, microbiota-derived signals can have context-dependent effects: metabolites such as SCFAs generally support barrier and immune homeostasis, whereas barrier failure permits other microbial products, including ADP-heptose and bacterial Toll-like receptor ligands, to promote inflammatory or clone-selective programs (Sections 2.1.3 and 2.2.1). Trained-immunity concepts therefore should not be extrapolated to a uniformly beneficial microbiota effect. Second, most evidence for durable microbiota-associated immune reprogramming in hematology derives from experimental models; because relevant exposures and thresholds in humans remain undefined, we do not attribute clinical outcomes to trained-immunity effects alone.

#### 2.1.3. Metabolic support and barrier protection

Microbiota-derived metabolites help maintain a low-inflammatory internal milieu compatible with stable hematopoiesis. SCFAs fuel colonocytes and promote immune homeostasis through GPR109A signaling and HDAC inhibition, enhancing Treg differentiation while limiting inflammatory cytokine production.^62^ When the epithelial barrier is breached, however, the same host–microbe interface can become pathogenic. ADP-heptose may enter the circulation and promote expansion of DNMT3A-mutant HSCs through ALPK1/NF-κB signaling, linking barrier disruption to clonal hematopoiesis.^25^ ^,^ ^64^

These mechanisms are not restricted to steady state. During stress hematopoiesis caused by infection or chemotherapy, microbial cues continue to influence compensatory blood-cell production. Depletion of SCFA-producing bacteria, for example, impairs recovery from chemotherapy-induced myelosuppression in mice,^14^ while transplantation studies associate sustained microbiota injury with impaired host defense and adverse outcomes.^59^ These observations provide a conceptual bridge between basal hematopoiesis and treatment-associated recovery without establishing a uniform causal effect in humans.

#### 2.1.4. Antibiotic-associated cytopenia: a clinically relevant link between antibiotic exposure, microbiota disruption, and hematopoiesis

Antibiotic-associated cytopenia provides a clinically relevant setting in which microbiota disruption can accompany abnormal hematopoiesis. In a multicenter pediatric study of prolonged antibiotic exposure, antibiotic-associated neutropenia was accompanied by reduced intestinal microbiome richness and depletion of Lachnospiraceae and associated metabolites.^65^ Murine studies argue against simple direct toxicity to progenitor cells: antibiotic-mediated microbiota depletion reproduces hematopoietic abnormalities seen in germ-free animals, and further antibiotic exposure in germ-free mice does not intensify suppression, supporting a microbiota-dependent mechanism in that model.^14^ ^,^ ^15^

Even in this relatively direct setting, clinical data remain too coarse to separate risks by antibiotic class, dose, duration, baseline microbiota, and host vulnerability. Stratified prospective studies are needed before microbiota preservation can be formally incorporated into hematology-specific antimicrobial stewardship.

### 2.2. Disease categories and microbiota associations

Disease-specific evidence is organized according to the five evidence strata defined in Section 1: Mendelian randomization, experimental models, prospective human cohorts, randomized human interventions, and cross-sectional studies. Within these strata, findings are interpreted using six cross-cutting functional pathways: short-chain fatty acids (SCFAs), bile-acid transformation, tryptophan metabolism, microbial translocation, epithelial barrier dysfunction, and innate immune signaling through pattern-recognition receptors. Because microbial composition varies with cohort, geography, diet, and treatment, individual taxa are treated primarily as examples of broader functional patterns.

#### 2.2.1. Benign and premalignant hematologic disorders

This group includes anemia, including thalassemia and sickle cell disease, immune thrombocytopenia (ITP), myelodysplastic syndromes (MDS), myeloproliferative neoplasms (MPN), clonal hematopoiesis of indeterminate potential (CHIP), and plasma-cell precursor states such as monoclonal gammopathy of undetermined significance (MGUS) and smoldering multiple myeloma (SMM). Evidence spans the five strata, but randomized human intervention data remain scarce. However, the biological context of microbiota–host interactions differs substantially across these disorders, and disease-specific findings should therefore not be generalized across the entire spectrum of benign and premalignant hematologic conditions.

##### 2.2.1.1. Mendelian randomization

MR provides human genetic evidence for a possible microbiota→host direction but does not establish mechanism. In ITP, selected microbial features and plasma metabolites, including sphingomyelin and the glucose-to-mannose ratio, have been associated with disease risk.^19^ These findings require independent replication.

In MDS, a bidirectional two-sample MR analysis of 456,348 participants identified eight microbial features associated with MDS risk, including prominent Blautia-related signals, and nominated immune phenotypes such as CD4+ T-cell proportion and CD127 expression on CD45RA+ non-regulatory CD4+ T cells as potential mediators.^22^ In MPN, Chen et al. combined the MiBioGen gut-microbiota GWAS (18,340 participants) with FinnGen data and identified associations between MPN risk and Coprococcus 3 and the Eubacterium hallii group.^26^ The latter analysis was limited by the number of MPN cases and the use of *p* < 5 × 10^−6^ for instrument selection. Overall, these findings are compatible with a potential directional relationship between microbial traits and hematologic disease, but should not be interpreted as definitive evidence that specific taxa initiate or drive disease.

##### 2.2.1.2. Experimental models

Experimental studies provide the strongest mechanistic evidence for microbial effects on hematopoiesis and immune regulation. Gut microbial metabolites such as 1,3-diaminopropane and reuterin can inhibit HIF-2α and reduce intestinal iron absorption; both metabolites ameliorated systemic iron overload in experimental models.^17^ These data establish a microbiota–metabolite–iron axis, but they should not be generalized to all anemia subtypes without disease-specific validation.

The strongest mechanistic evidence links intestinal barrier injury to clonal hematopoiesis. In mice transplanted with Dnmt3a-deficient bone marrow, dextran sulfate sodium-induced injury or aging selectively expanded mutant hematopoietic stem cells; this effect was suppressed by antibiotics targeting Gram-negative bacteria and reproduced by microbiota transfer.^25^ Increased intestinal permeability allowed ADP-heptose to enter the circulation, activate the ALPK1–TIFA–NF-κB pathway, and promote expansion of preleukemic cells. Concordant human observations include circulating ADP-heptose in older individuals and patients with MDS, increased ALPK1 expression in CD34+ cells from patients with MDS, and enhanced expansion of human MDS cells after ADP-heptose exposure in xenotransplantation models. A parallel TLR2/IL-6 pathway can promote expansion of TET2-deficient preleukemic stem cells.^66^

Germ-free and microbiota-depleted models further show that commensal microbes contribute to normal hematopoietic and immune function. Germ-free mice have reduced granulocyte–monocyte progenitors and colony-forming capacity, increased susceptibility to Listeria monocytogenes, and impaired resistance that is restored by recolonization.^16^ However, related models indicate that myeloid proliferation can also occur independently of commensal bacteria and may instead be driven by autoreactive T cells.^67^ Yan et al. further suggested that antibiotic-associated neutropenia is more likely indirect and microbiota-mediated because drug concentrations suppressing progenitor cells in vitro exceed those achieved in vivo.^68^

MPN models provide complementary evidence of intestinal involvement. Zhu et al. reported greater intestinal inflammation, reduced tight-junction protein expression, and activation of the caspase-1/IL-1β pathway in an MPLW515L transplantation model.^27^ However, Humblet-Baron et al. showed that impaired dendritic-cell and CD4+ T-cell interactions can independently produce a murine myeloproliferative phenotype, indicating that not all inflammatory pathways are necessarily microbiota-dependent.^69^ Together, these findings support intestinal inflammation as part of MPN biology while cautioning against attributing myeloproliferation to the microbiota alone.

Animal microbiota-transfer studies provide experimental rather than human intervention evidence. In transgenic sickle cell disease models, FMT from healthy donors reduced chronic pain, increased protective taxa including Akkermansia muciniphila, altered bilirubin-related metabolism, and reduced pronociceptive metabolites acting through TRPM2; reverse transfer reproduced persistent pain in wild-type mice.^70^ Engineered nanoparticles have also been proposed as oral iron formulations that restore gut microbial composition and improve anemia-related biomarkers.^71^ ^,^ ^72^

##### 2.2.1.3. Prospective cohorts

Prospective human evidence remains limited but provides temporal information. In ITP, baseline fecal metagenomic profiles were evaluated in patients who subsequently received corticosteroid therapy, with 152 treated participants completing a four-week efficacy assessment. Baseline microbiome features contributed to prediction of corticosteroid response, providing prospective predictive evidence while not establishing a causal microbiota mechanism.^20^

In MDS, interpretation remains constrained by small samples, marked clinical heterogeneity, and a lack of pre-diagnostic baseline sampling.^73^ These limitations make it difficult to determine whether observed microbial changes precede disease or arise from established disease and its associated exposures.

##### 2.2.1.4. Randomized human interventions

Randomized intervention evidence remains sparse. Dietary fiber can alter microbial fermentation and SCFA production.^74^ In a prospective randomized placebo-controlled study of maintenance-hemodialysis patients with renal anemia, eight weeks of soluble dietary fiber increased hemoglobin and iron indices while altering gut microbiota composition and serum SCFAs.^18^ This is a clinically informative intervention signal, but it remains confined to renal anemia and does not establish general efficacy across anemia subtypes.

In MPN, Avelar-Barragan et al. randomized 28 patients to Mediterranean-diet counseling (*n* = 15) or a standard American diet (*n* = 13) for 15 weeks. No significant diet-associated change in the microbiota was observed, although some genera correlated with inflammatory cytokines.^28^

No randomized human trial in benign or premalignant hematologic disease has yet demonstrated that targeted microbiota modification improves a clinically meaningful hematologic outcome; current trials primarily demonstrate changes in microbial composition, inflammatory markers, or hematologic surrogate endpoints.

##### 2.2.1.5. Cross-sectional studies

Cross-sectional studies provide the broadest evidence for disease-associated dysbiosis but cannot establish temporal direction. In non-transfusion-dependent *β*-thalassemia/HbE, reduced diversity and lower abundance of SCFA-producing taxa have been associated with iron overload.^75^ In transfusion-dependent *β*-thalassemia, altered microbial metabolites—including higher trimethylamine-*N*-oxide and lower fecal SCFAs—have been associated with iron burden, inflammation, and renal tubular dysfunction.^76^ ^,^ ^77^ These relationships are inherently bidirectional: host iron availability can reshape the gut community, while bacteria compete for iron through uptake systems and siderophore-mediated scavenging.^78^ Thus, dysbiosis and iron overload cannot be assigned a unidirectional causal relationship from cross-sectional data alone.

In MDS, gut and peripheral-blood microbial communities differ from those of healthy controls, with community structure associated with disease severity and clinical features.^24^ In ITP, cross-sectional microbiome and metabolome studies report dysbiosis and metabolite changes associated with platelet count, while mechanistic links to immune tolerance remain inferential.^21^ ^,^ ^79^

In MPN, Eickhardt-Dalbøge et al. found greater richness, distinct community composition, and lower Faecalibacterium abundance in essential thrombocythemia than in healthy controls (8% vs 15%, *p* < 0.001), with stronger differences in JAK2V617F-positive patients.^29^ Exploratory studies have also detected microbial or viral sequences in blood or bone marrow in myeloid malignancies, but these findings do not establish a gut origin; circulating microbial sequences have been associated with disease subtype and prognosis,^80^ and Merkel cell polyomavirus DNA was more frequently detected in MPN marrow than in controls.^81^ These observations remain associative and should not be interpreted as evidence that intestinal microbes directly drive MPN.

#### 2.2.2. Hematologic malignancies

The same evidence framework can be applied to leukemia, lymphoma, multiple myeloma (MM), and chronic lymphocytic leukemia (CLL), although the biological context and clinical relevance of microbiota–host interactions vary substantially across diseases and treatment settings. Recurring functional themes include loss of beneficial microbial activity, epithelial barrier dysfunction, microbial translocation, altered metabolites, and microbiota-dependent effects on treatment.

##### 2.2.2.1. Mendelian randomization

Human genetic evidence remains limited. In diffuse large B-cell lymphoma (DLBCL), MR has implicated Ruminococcaceae UCG-002 as a potential risk factor, possibly through inflammatory cytokines such as CXCL9/MIG.^34^ This supports a possible microbiota→host direction but does not establish mechanism. Comparable MR evidence remains limited for leukemia, MM, and CLL.

##### 2.2.2.2. Experimental models

In genetically predisposed pre-B-cell ALL models, early-life antibiotic exposure can promote leukemia, suggesting that loss of commensal protection may contribute to leukemogenesis.^30^ In the Eμ-TCL1 CLL model, reduced microbial diversity under high-hygiene conditions accelerates disease progression, supporting a protective role for microbial richness.^42^

Microbial products and metabolites can modify tumor microenvironments. Lipopolysaccharide activates stromal and osteoclast-associated signaling and increases IL-6, VEGF, and MMP-9, whereas indoxyl sulfate may promote MM progression through AhR-dependent pathways.^82^ Conversely, microbiota-derived butyrate can exert anti-MM effects on the bone-marrow microenvironment.^83^ In NK/T-cell lymphoma, butyrate-producing taxa and butyrate can suppress tumor growth through JAK–STAT signaling.^35^ ^,^ ^84^

Cutaneous T-cell lymphoma provides a tissue-specific, non-gut example of host–microbe interaction. In mycosis fungoides, a subgroup of patients showed lesional dysbiosis with Staphylococcus aureus outgrowth; plaque-derived S. aureus also showed increased expression of the virulence factor spa, which may activate NF-κB signaling.^85^ This finding illustrates tissue-specific microbial biology and should not be extrapolated directly to the intestinal microbiome.

In CLL, microbiome diversity itself may be related to disease course. In a cohort of 59 patients, lower gut microbial diversity was associated with more advanced or aggressive disease, and in Eμ-TCL1 mice, high-hygiene conditions accompanied by reduced microbiome diversity accelerated CLL progression.^42^ Given the antigen-driven biology of CLL and the frequent use of stereotyped B-cell receptors,^86^ conserved microbial antigens remain a plausible but unproven contributor to clonal selection. Current evidence does not establish a specific microbial antigen or a direct gut-barrier mechanism in human CLL.

The microbiota may also modify treatment directly. Intestinal bacterial O-demethylation of etoposide generates metabolites with greater genotoxic activity and alters systemic exposure to the parent drug.^87^

##### 2.2.2.3. Prospective cohorts

Prospective and longitudinal studies are most informative when microbial sampling precedes the clinical outcome of interest. In pediatric ALL, microbiota changes from diagnosis through induction were associated with prolonged neutropenia and delayed neutrophil reconstitution.^32^ In AML, longitudinal sampling during induction chemotherapy has documented substantial treatment-associated shifts in microbiota composition that can persist after hospital discharge.^33^ These temporal data are valuable for treatment-associated effects but do not establish a pre-disease causal microbiome.

HSCT provides an important prospective setting. Allogeneic HSCT remains potentially curative for leukemia and lymphoma, but GVHD, infection, and relapse remain major causes of treatment failure.^88^ Longitudinal transplant studies have linked peri-transplant microbiome and metabolome disruption with GVHD and other adverse clinical outcomes.^45^ ^,^ ^47^ ^,^ ^89^ Persistent dysbiosis can remain detectable years after allo-HSCT and has been associated with late complications.^90^ Expansion or translocation of oral bacteria and altered microbial bile-acid metabolism have also been linked to transplant-related complications.^46^ ^,^ ^91^ ^,^ ^92^ These associations provide temporal information but remain strongly confounded by conditioning, antimicrobial exposure, nutrition, and disease severity.

##### 2.2.2.4. Randomized human interventions

Randomized microbiota-directed studies remain limited, but the evidence base has progressed beyond purely observational studies. In a randomized, double-blind, placebo-controlled phase II trial involving allogeneic HCT recipients and patients with AML receiving induction chemotherapy, oral encapsulated third-party FMT improved post-antibiotic recovery of microbial diversity and restored several depleted obligate anaerobes, but did not significantly reduce the rate of infections during the study period.^48^ Thus, the trial demonstrated ecological restoration without establishing improvement in a clinically meaningful infectious outcome.

##### 2.2.2.5. Cross-sectional studies

Cross-sectional evidence remains useful for describing disease-associated ecology but cannot establish temporal direction. At diagnosis, pediatric ALL patients show gut-microbiota profiles that differ from healthy controls.^31^ In treatment-naïve DLBCL, lower diversity and Enterobacteriaceae enrichment have been associated with febrile neutropenia, infection risk, treatment outcome, and shorter progression-free survival.^37^ ^,^ ^38^ In MM, observational cohorts have linked SCFA-producing bacteria and circulating SCFAs with disease state, treatment response, and minimal residual disease, while mechanistic experiments support antiproliferative effects of selected SCFAs.^40^ ^,^ ^82^ In CLL, cross-sectional analyses have revealed associations of disease state and medication exposure with gut microbiome composition and diversity.^43^ These findings are hypothesis-generating unless supported by prospective or experimental evidence.

A conference abstract has also reported associations between gut-microbiota diversity and follicular-lymphoma disease features.^39^ Because these findings have not yet been validated in a full peer-reviewed report, they should be considered preliminary. The broader heterogeneity across lymphoma subtypes reinforces the need for subtype-specific rather than pan-lymphoma analyses.

Treatment is a major confounder across these diseases. Chemotherapy and antibacterial prophylaxis can alter the microbiota independently of malignancy, whereas established disease can itself affect immunity, nutrition, epithelial integrity, and metabolism. Findings obtained after treatment should therefore not be interpreted as evidence of a pre-disease microbial cause.

### 2.3. Bidirectional interactions and reverse causality

The relationship between the microbiota and hematologic disease is generally bidirectional rather than unidirectional. Microbial signals may influence hematopoiesis, clonal selection, inflammation, and treatment response, while disease, therapy, antimicrobial exposure, altered immunity, and mutant hematopoietic cells can simultaneously reshape the intestinal environment. The resulting interaction is therefore better conceptualized as a dynamic feedback system than as a simple linear pathway from dysbiosis to disease.

Treatment is a major source of reverse causality. Induction chemotherapy, hypomethylating agents, and broad-spectrum antibacterial prophylaxis can substantially reshape microbial diversity and community structure.^32^ ^,^ ^33^ ^,^ ^59^ In MDS, glutamine-synthesis and tryptophan-metabolism pathways are prominent at diagnosis and attenuate after hypomethylating-agent therapy,^23^ illustrating treatment-associated functional change rather than direct microbiota intervention. Consequently, post-treatment microbiome differences cannot be interpreted as evidence that dysbiosis preceded disease. For leukemia and related myeloid disorders, the most informative window for causal inference is before diagnosis or before the first treatment cycle, although such samples are rarely available.

The mutant clone can also impair host defense. CHIP is associated with an increased risk of bacterial pneumonia, while Tet2-deficient myeloid cells exhibit impaired neutrophil migration, pathogen uptake, and neutrophil extracellular trap formation, supporting a defect in innate antibacterial defense.^93^ Conversely, infection burden predicts incident CHIP (OR 1.41, *p* = 0.03; OR 1.83, *p* = 0.008 for large clones).^94^ Together, these findings support a bidirectional and potentially self-reinforcing relationship between clonal hematopoiesis and infectious exposure.

MPN provides a similar example. JAK2V617F-positive patients have distinct microbial community structure, while experimental models demonstrate intestinal inflammation and barrier abnormalities.^27^ ^,^ ^29^ At the same time, impaired dendritic-cell and CD4+ T-cell interactions can independently produce a myeloproliferative phenotype in mice,^69^ indicating that not all inflammatory pathways are necessarily microbiota-dependent. Observational data therefore cannot determine whether dysbiosis is upstream, downstream, or part of a mutually reinforcing process.

The microbiota can additionally modify treatment through drug metabolism. Bacterial O-demethylation of etoposide generates metabolites with greater genotoxicity and alters exposure to the parent drug.^87^ Thus, treatment can reshape the microbiota while the microbiota simultaneously modifies drug exposure.

Accordingly, findings throughout this review are interpreted according to the strongest inference permitted by the study design: cross-sectional studies support association; prospective cohorts provide temporal evidence; MR informs potential causal direction; experimental models provide biological and mechanistic support; and randomized interventions test whether microbiota modification alters outcomes. This distinction is particularly important for CHIP, MPN, leukemia, and HSCT, where disease, treatment, inflammation, antimicrobial exposure, and microbial signals may form a self-reinforcing loop.

The major research priority is therefore to move beyond additional taxonomic associations toward longitudinal human sampling, functional microbial measurements, mechanistic validation, and randomized interventions linked to clinically meaningful hematologic outcomes.

## 3. Microbiota-oriented immune regulation in hematologic diseases

Systemic immune programs influence tissue physiology and hematopoietic homeostasis broadly.^95^ Within this framework, gut microbiota-derived metabolites and microbe-associated molecular patterns can shape immune-cell states with downstream effects on marrow hematopoiesis and disease biology.^8^ ^,^ ^9^ Because T cells, B cells, and myeloid cells form key nodes of adaptive and innate immune remodeling, this section follows these three cellular layers (Figure 2). To complement this overview, Tables 2–4 summarize representative microbiota-guided mechanisms involving T cells, B cells, and myeloid cells, respectively.

**Figure 2.**
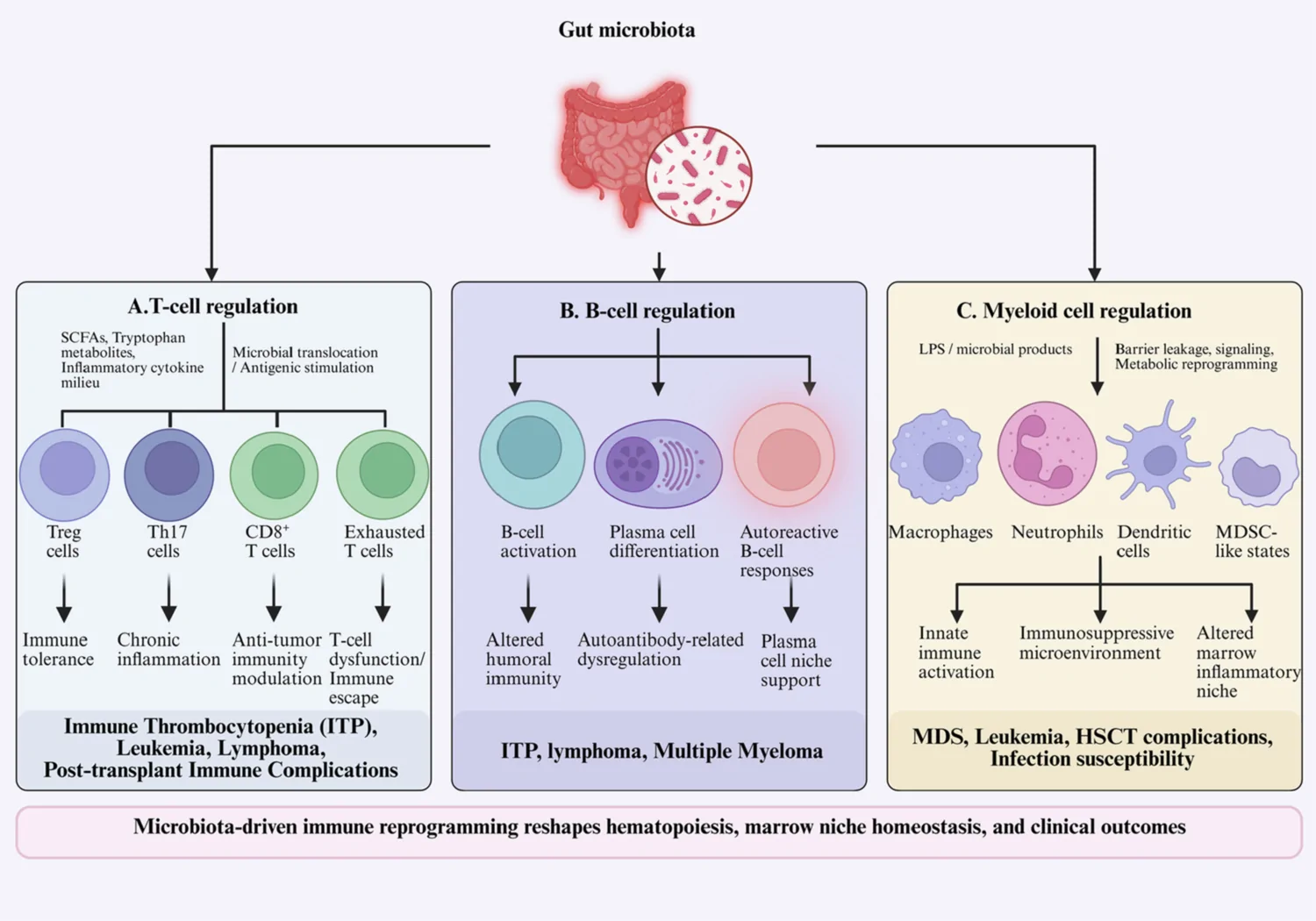
Layered immune-regulatory network linking the gut microbiota to hematologic diseases. Microbial signals and metabolites act on three principal immune compartments: T cells, B cells, and myeloid cells. At the T-cell level, SCFAs, tryptophan metabolites, and inflammatory cytokine milieus modulate Treg cells, Th17 cells, CD8+ T cells, and exhausted T cells, influencing immune tolerance, chronic inflammation, antitumor immunity, and immune escape. At the B-cell level, microbiota-related cues can drive B-cell activation, plasma-cell differentiation, and autoreactive B-cell responses, thereby shaping humoral immunity, autoantibody-associated dysregulation, and plasma-cell niches. At the myeloid level, microbial products such as LPS and barrier-disruption signals can remodel macrophages, neutrophils, dendritic cells, and MDSC-like states, generating inflammatory or immunosuppressive marrow microenvironments. Together, these programs affect disease initiation, progression, and therapeutic response.

### 3.1. Microbiota–T-cell crosstalk

Gut microbes and their metabolites promote peripheral differentiation and functional specialization of Treg cells. Separately, marrow-resident Treg cells home through CXCR4 and help maintain HSC quiescence by converting extracellular ATP to adenosine via the CD39/CD73 axis.^96^ These observations provide a plausible route by which microbiota-dependent changes in Treg abundance or function could influence hematopoietic niches, but direct evidence that microbial signals regulate marrow Treg homing in hematologic disease remains lacking. Microbial metabolites also set the balance between regulatory and effector T-cell programs: SCFAs restrain Th17 differentiation and support Treg induction, whereas tryptophan metabolites acting through the aryl hydrocarbon receptor sustain IL-22-dependent epithelial repair and thereby limit chronic inflammatory signals that drive T-cell exhaustion.^61^ Propionate can also induce immunogenic cell death through ACSL4-dependent ferroptosis, enhancing dendritic-cell maturation and CD8+ T-cell-mediated antileukemic immunity.^97^

Human evidence consistent with microbiota–T-cell coupling comes from allo-HSCT, where azithromycin exposure was associated with shifts in gut enterotypes, relapse-related microbial and metabolic signatures, and circulating exhausted T-cell states.^98^ These observations link antibiotic-driven ecosystem change with systemic T-cell phenotypes, but they remain associative at the level of individual microbial taxa and metabolites. Representative mechanisms underlying microbiota-guided regulation of T-cell immunity are summarized in Table 2. What remains unresolved in this compartment is specificity: the same metabolites can act on Treg, Th17, CD8+ and exhausted T cells, and no study in hematologic disease has yet shown that a defined microbial product shifts this balance in a predictable direction in patients.

**Table 2.** Microbiota-guided T-cell immune regulation.

Immune-cell class	Cell/molecule	Microbial or metabolic signal	Core mechanism	Related disease context	References
T cells	Treg and Th17 cells	SCFAs and other microbial metabolites	SCFAs and related microbial metabolites modulate Treg differen tiation and the Treg–Th17 balance through GPCR- and HDAC-dependent pathways; direct control of marrow Treg homing by gut microbiota remains unproven	Systemic immune homeostasis; ITP/MM contexts	[61,62]
	Dendritic cells and CD8+ T cells	Propionate	ACSL4/ICD signaling induces DAMP release, drives dendritic-cell maturation, and activates CD8 + T-cell–mediated antileukemic adaptive immunity	Leukemia	[97]
	inflammatory T cells	Oral-microbiota dysbiosis	Oral pathobionts reach cervical lymph nodes, activate antigen-presenting cells, expand inflammatory T cells, and promote ectopic intestinal colonization	cGVHD	[99]

### 3.2. Microbiota–B-cell crosstalk

Prospective post-allo-HSCT sampling showed that patients who later developed cGVHD had early expansion of IgA-dominant plasmablasts, many of which produced antibodies reactive with commensal bacteria.^100^ In ITP, microbial contributions to autoimmunity remain incompletely defined; molecular mimicry is best established in infection-associated ITP, particularly Helicobacter pylori-associated disease, rather than as a general consequence of gut dysbiosis.^101^ ^,^ ^102^ More broadly, NF-κB remains a central signaling hub downstream of microbial and inflammatory cues in B cells, although this general biology should not be conflated with disease-specific microbiota causality.^10^ ^,^ ^103^

The clearest clinically actionable B-cell paradigm is gastric MALT lymphoma, in which chronic Helicobacter pylori-driven antigenic stimulation contributes to lymphomagenesis and eradication therapy can induce durable remission in many early-stage cases.^102^ ^,^ ^104^ Together with the cGVHD and ITP examples above, this illustrates that microbiota–B-cell interactions range from commensal-reactive mucosal antibody responses to infection-associated autoimmunity and chronic antigen-driven lymphoma. Representative mechanisms are summarized in Table 3.

**Table 3.** Microbiota-guided B-cell immune regulation.

Immune-cell class	Cell/molecule	Microbial or metabolic signal	Core mechanism	Related disease context	References
B cells	MALT B-cell clones	Helicobacter pylori antigens	Chronic antigen-driven B-cell stimulation contributes to gastric MALT lymphomagenesis; eradication can induce durable remission in early-stage disease	Gastric MALT lymphoma	[102,104]
	Plasmablasts; IgA	Commensal-reactive mucosal antibody responses after allo-HSCT	Early expansion of IgA-dominant plasmablasts, many producing commensal-reactive antibodies, precedes and predicts subsequent cGVHD	cGVHD	[100]
	Autoreactive B cells; antiplatelet autoantibodies	Microbial antigens, particularly in infection-associated ITP	Molecular mimicry and antigenic cross-reactivity can promote antiplatelet autoantibodies; H. pylori-associated ITP provides the clearest microbial paradigm	ITP	[101,102]

### 3.3. Microbiota–myeloid immune crosstalk

In AML, fecal and plasma chenodeoxycholic acid levels are reduced, positively correlate with gut microbiota diversity, and inversely correlate with poor prognosis. Functionally, chenodeoxycholic acid promotes ROS/p38 MAPK/DGAT1-mediated lipid-droplet accumulation and lipid peroxidation in AML cells while inhibiting M2 macrophage polarization, thereby restraining AML progression.^105^ Dysbiosis can also cooperate with innate immune defects: loss of TRAF3 in myeloid cells causes microbial disruption, microbial translocation, and systemic antibacterial IgG responses that eventually transform bacteria-reactive B-cell clones into lymphoma.^106^

Myeloid regulation is strongly context dependent. In DLBCL, treatment-naïve patients display gut dysbiosis characterized by Enterobacteriaceae enrichment, antibiotic-resistance-related microbial functions, inflammatory cytokine associations, febrile neutropenia, and shorter progression-free survival.^37^ In childhood ALL, loss of selected commensal taxa and Enterococcus overgrowth during induction are associated with prolonged neutropenia and higher CXCL1/CXCL8 levels, consistent with altered neutrophil recovery and trafficking; causal direction remains unresolved.^32^ These observations support clinically relevant links between microbiota state and myeloid outcomes, but they do not establish that microbiota-targeted intervention will correct neutropenia. Representative mechanisms are summarized in Table 4.

**Table 4.** Microbiota-guided regulation of myeloid immune cells.

Immune-cell class	Cell/molecule	Microbial or metabolic signal	Core mechanism	Related disease context	References
Myeloid cells	M2 macrophages	Chenodeoxycholic acid; microbiota diversity	CDCA promotes ROS/p38 MAPK/DGAT1 signaling, lipid-droplet accumulation, and lipid peroxidation while suppressing M2 polarization, thereby inhibiting AML progression	AML	[105]
	Macrophages; TRAF3	Dysbiosis and translocation of specific pathobionts	Loss of TRAF3 in myeloid cells causes innate immune defects, dysbiosis, microbial translocation, and systemic antibacterial IgG responses, driving malignant transformation of bacteria-reactive B-cell clones	B-cell lymphoma	[106]
	Neutrophils; CXCL1/CXCL8	Treatment-associated microbiota change; Enterococcus overgrowth	Loss of selected commensals and Enterococcus overgrowth are associated with higher CXCL1/CXCL8 and prolonged neutropenia during induction; causal direction remains unresolved	Childhood ALL; induction-related neutropenia	[32]

## 4. Translational applications of the gut microbiota in hematologic diseases: diagnosis, risk stratification, and microbiota-directed intervention

The clinical translation of gut microbiota research in hematologic diseases can be considered across three closely related areas: diagnosis, risk stratification, and microbiota-directed intervention. Because the composition of a healthy microbiota varies with geography, diet, lifestyle, medication exposure, and population ancestry, clinically useful microbiome-based approaches will require population-specific reference ranges.^107^ In hematologic cancers, accumulating pharmacomicrobiomic evidence indicates that gut microbial communities and metabolites can modify drug chemistry, immune tone, therapeutic efficacy, and toxicity.^108^ Figure 3 summarizes how microbiome discoveries in hematologic disease may progress from association studies to mechanistic validation, biomarker development, intervention design, and clinical implementation.

**Figure 3.**
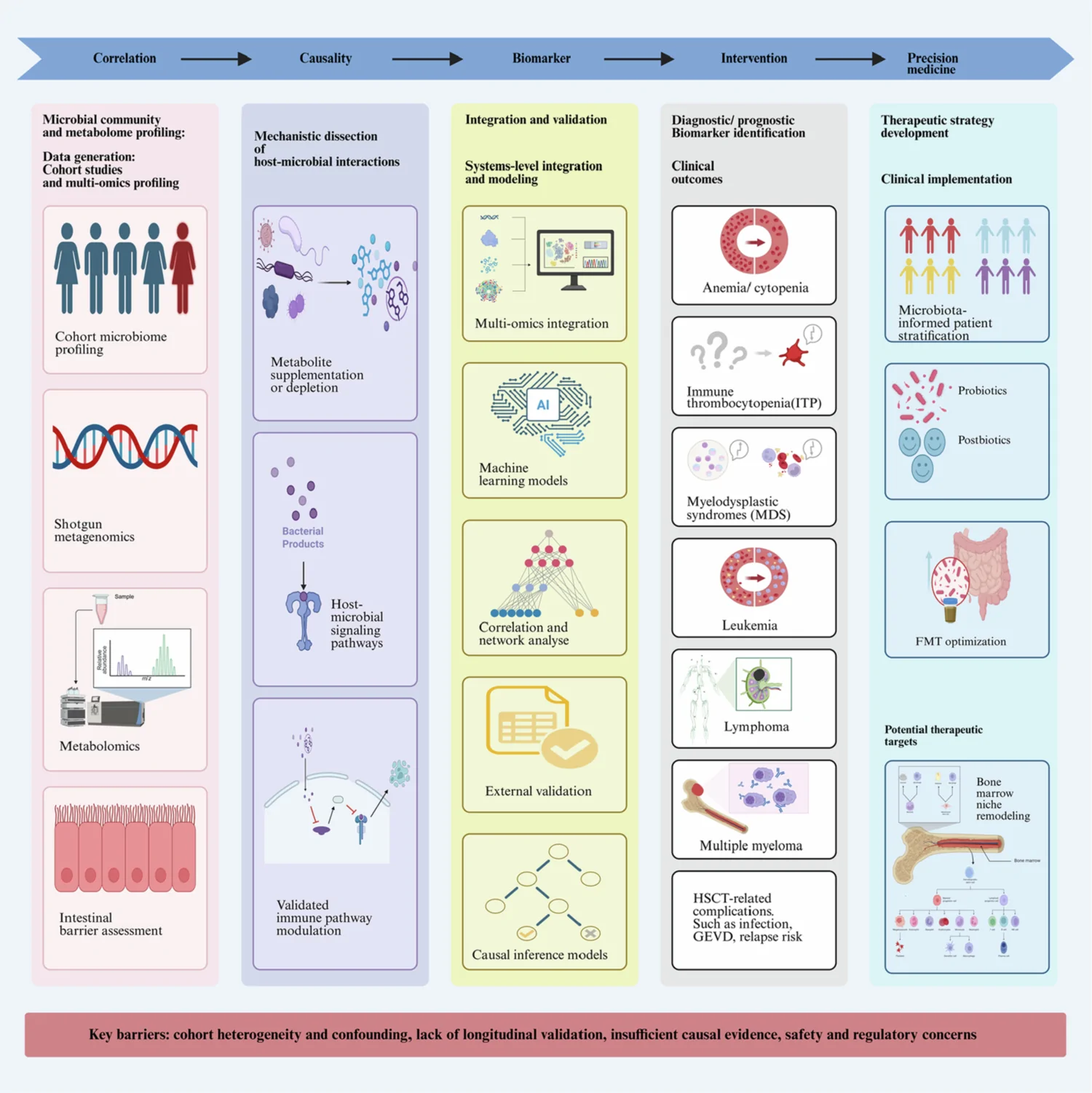
Translational pipeline of gut microbiota research in hematologic diseases. The pipeline moves from microbial-community and metabolome profiling to mechanistic dissection of host–microbial interactions, systems-level integration, external validation, biomarker identification, and therapeutic development with clinical implementation. Major barriers include cohort heterogeneity and confounding, limited longitudinal validation, weak causal evidence in some disease settings, and unresolved safety and regulatory issues.

### 4.1. Microbiota-based diagnosis

Specific microbiota features show diagnostic promise in hematologic disease. In ITP, combinations involving Weissella, Streptococcus anginosus, and selected ceramide signatures have been reported as candidate non-invasive biomarkers.^21^ Gastric MALT lymphoma provides a well-established example in which Helicobacter pylori is both mechanistically involved in disease and clinically actionable.^102^ Circulating microbial sequences represent another exploratory biomarker class: in myeloid malignancies, circulating microbial content has been associated with disease subtype and patient outcome, but contamination control, source attribution, and analytical standardization remain major obstacles.^80^

Circulating cell-free microbial DNA may become another diagnostic frontier. Compared with stool profiling, plasma microbial DNA may better reflect systemic microbial translocation and could track clinically relevant barrier disruption. Standardization remains a major obstacle, however, and reproducibility across disease types, sequencing platforms, and analytical pipelines has not yet been established.

Taken together, these findings support the potential of the gut microbiota as a source of non-invasive biomarkers for hematologic diseases. However, diagnostic application will require population-adapted reference ranges, standardized analytical methods, and prospective validation before microbiota-based testing can be incorporated into routine clinical practice.

### 4.2. Risk stratification and treatment-response prediction

Beyond disease detection, microbiota-derived features may help stratify patients by the risk of treatment-related complications and may help predict response to specific therapies. In AML induction, higher baseline stool diversity is associated with lower infectious risk, whereas prolonged carbapenem exposure is associated with lower diversity at neutrophil recovery and a higher risk of subsequent infection; antibiotic-induced perturbation also varies substantially according to baseline microbiota.^109^ ^,^ ^110^ In HSCT, peri-transplant microbiome and metabolome disruption and persistent dysbiosis have been associated with GVHD, disease progression, survival, and late complications.^45^ ^,^ ^47^ ^,^ ^89^ ^,^ ^90^ These findings support microbiome-informed risk assessment but do not yet define validated clinical thresholds.

Microbiota composition may also contribute to prediction of treatment response. In ITP, baseline microbial features have been incorporated into microbiota-based prediction models for corticosteroid responsiveness.^20^ During CAR-T therapy, baseline microbiome composition and diversity, as well as antibiotic exposure around treatment, have been associated with cytokine release syndrome, neurotoxicity, treatment efficacy, and survival.^49^ ^,^ ^50^ ^,^ ^111^

Together, these findings indicate that microbiota-derived features may complement conventional clinical factors in stratifying patients by risk or expected treatment response. Despite these promising findings, microbiota-based prediction is not yet ready for routine clinical use. Most models lack multicenter external validation, and a large proportion of interindividual variation in microbiota composition remains unexplained by currently measured factors. Accordingly, microbiota-based measures should currently complement, rather than replace, established clinical and laboratory predictors.

### 4.3. Microbiota-directed intervention and clinical translation

The microbiota is increasingly viewed not only as a biomarker but also as a modifiable determinant of treatment efficacy and toxicity. Cyclophosphamide provides one of the best-characterized examples: treatment-related microbial perturbation can permit translocation of selected Gram-positive bacteria to secondary lymphoid organs, enhance pTh17 and memory Th1 responses, and strengthen antitumor immunity.^112^ Conversely, germ-free conditions attenuate this effect, indicating that the relationship between microbial disturbance and treatment response is context dependent rather than uniformly detrimental. More broadly, gut microbiota can influence chemotherapy efficacy and toxicity through immunomodulation, microbial translocation, enzymatic transformation, and ecological disruption.^113^

#### 4.3.1. Microbiota-directed strategies to preserve or restore the microbiota

These observations have encouraged strategies aimed at preserving the microbiota during treatment. When clinically feasible, narrower-spectrum antibiotics may reduce unintended loss of beneficial anaerobic bacteria. In a multicenter CAR-T study, exposure to anaerobe-targeting broad-spectrum antibiotics—particularly piperacillin/tazobactam, meropenem, or imipenem/cilastatin—during the four weeks before infusion was associated with worse survival and increased neurotoxicity.^49^ Because antibiotic indication and infection severity are important confounders, these observational data support antibiotic stewardship rather than avoidance of clinically necessary therapy.

Lifestyle-based microbiota modulation has also been explored. In a small pilot study of patients with multiple myeloma in remission, six weeks of Nordic walking combined with time-restricted eating increased microbial biodiversity and shifted community composition toward that observed in healthy controls; however, the study did not establish a clinically meaningful hematologic benefit.^114^

Direct restoration of the microbiota offers another potential therapeutic approach. In AML, autologous FMT has been reported to restore microbial diversity, increase beneficial Lachnospiraceae and Ruminococcaceae, and reduce antibiotic-resistance gene burden.^52^ In aging models, FMT from young donors partly reversed age-related HSC defects, while Lachnospiraceae and tryptophan-associated metabolites improved measures of HSC abundance, function, and engraftment capacity.^115^ In HSCT, Clostridium butyricum MIYAIRI 588 administration has been associated with maintenance of early microbial diversity and lower relative abundance of Enterococcus and Bacteroides,^53^ whereas a single-patient GVHD study documented dynamic remodeling of the bacteriome, mycobiome, and virome after serial FMT.^116^ A separate myelofibrosis case report described control of recurrent sepsis after washed microbiota transplantation, but neither single-patient observation can establish efficacy or generalizability.^117^

#### 4.3.2. Microbiota-derived metabolites as therapeutic agents

Microbiota-derived metabolites provide another potential route for therapeutic intervention. In MM, urolithin A has shown antimyeloma activity and enhanced bortezomib effects in experimental systems, including resistant cells.^41^ Nisin produced by Lactococcus lactis and combinations involving urolithin B and vincristine have shown synergistic antiproliferative and pro-apoptotic effects against lymphoma cells in vitro.^118^ More broadly, microbial metabolism and immunomodulation can alter anticancer-drug pharmacokinetics and pharmacodynamics, although hematology-specific mechanisms remain incompletely defined.^119^ These approaches remain preclinical unless supported by human pharmacokinetic, safety, and efficacy data.

#### 4.3.3. Safety and implementation considerations

The clinical translation of these approaches requires particular attention to safety and reproducibility. Because patients with hematologic malignancies are often profoundly immunosuppressed, FMT requires stringent donor screening for latent viral infections, multidrug-resistant organisms, potentially oncogenic microorganisms, and relevant inflammatory or autoimmune conditions.^120^ Small autologous-FMT feasibility studies in HSCT also illustrate substantial logistical and patient-selection constraints; in one study, four recipients received autologous FMT by retention enema.^121^ Alternative formulations, including encapsulated donor products as used in randomized trials,^48^ may improve feasibility, but formulation-specific safety, engraftment, and efficacy require prospective evaluation.

Overall, microbiota-directed therapy has moved beyond proof of biological association, but clinical implementation remains limited by incomplete causal evidence, heterogeneous study populations, inconsistent microbiome testing, limited long-term safety data, and inadequate multicenter validation. Standardized methods and prospective clinical studies will be essential to determine which microbiota-based biomarkers and interventions provide reproducible clinical benefit.

## 5. Conclusions and Perspectives

### 5.1. Conclusions

Taken together, the reviewed literature supports a microbiota–immune–hematology axis as an important determinant of hematologic homeostasis and disease evolution. Through microbial metabolites and MAMPs, the gut microbiota contributes to HSPC lineage commitment, immune-cell polarization, inflammatory microenvironment formation, therapeutic response, epigenetic remodeling, metabolic regulation, and barrier maintenance.

The strongest near-term translational opportunity lies in prevention and management of HSCT-related complications, where prospective cohorts and randomized FMT studies already exist. ITP and selected leukemia settings have stronger genetic or experimental causal support than many cross-sectional disease areas but still lack microbiota-directed interventional confirmation. Other areas, including anemia subtype stratification, human pharmacokinetics of urolithin A in MM, and several lymphoma or CLL contexts, remain driven mainly by association or hypothesis-generating evidence.

This distinction should guide research priorities. Resources are better directed toward questions with a plausible mechanism but missing interventional evidence—for example, microbiota-based randomized studies in ITP—than toward further descriptive association studies in settings where the same observations have already been repeatedly documented.

### 5.2. Perspectives

Several priorities follow from this synthesis. First, the field needs harmonized standards for sampling time, DNA extraction, 16S variable-region selection, and minimum sequencing depth for shotgun metagenomics; without these, cross-study comparison and meta-analysis will remain fragile. Second, causal designs should be favored over additional cross-sectional description. For ITP, DLBCL, MDS, and related diseases, the next step should be longitudinal cohorts with pre-diagnostic sampling, broader MR analyses, and mechanistic validation that generalizes beyond a single model. Third, interventional studies should use disease-specific clinical endpoints, such as progression-free survival, complete remission, or GVHD incidence, rather than microbial shifts alone.

For the disease settings discussed in Section 2, the decisive missing design is pre-diagnostic or pre-treatment sampling within longitudinal cohorts, which would allow disease-driven and treatment-driven microbial differences to be separated. Equally important is triangulation across Mendelian randomization, microbiota-depletion or colonization models, molecular mechanism, prospective human cohorts, and randomized interventions, rather than citing these evidence strata independently of one another (Sections 2.2.1, 2.2.2, and 2.3).

Mechanistically, future work should combine germ-free models, defined-colonization systems, and gene-editing approaches to determine how specific taxa or metabolites regulate HSC fate, malignant clonal evolution, and treatment response through immune receptors, epigenetic programming, and metabolic rewiring. The route by which intestinal microbial signals reach the bone marrow as a remote target organ deserves particular attention.

Future translational studies should prioritize interventions supported by convergent evidence across longitudinal human cohorts, functional microbial measurements, and mechanistic models, and should evaluate clinically meaningful hematologic endpoints rather than microbiome changes alone. FMT, probiotics, postbiotics, and metabolite-based approaches remain promising, but their optimal timing, dose, duration, patient selection, and long-term safety—particularly in immunocompromised populations—require prospective evaluation. Ultimately, integrating microbial function with host immune and hematopoietic phenotypes, while explicitly accounting for bidirectional causality and treatment-related perturbations, will be essential for determining when the gut microbiota represents a clinically actionable component of hematologic disease rather than merely a correlate of disease state.

Population specificity also warrants emphasis. Gut microbial architecture differs substantially between Chinese and Western populations, and conclusions drawn from North American or European cohorts cannot be directly transferred to Chinese clinical practice without local validation. Large prospective China-based cohorts will therefore be essential for closing the evidence gap and for designing microbiota-targeted interventions appropriate to local populations.

## Data Availability

Data sharing is not applicable to this article because no new data were created or analyzed in this review.
